# Editorial: Novel Vaccine Technologies in Animal Health

**DOI:** 10.3389/fvets.2022.866908

**Published:** 2022-02-25

**Authors:** Vasilis C. Pliasas, George C. Fthenakis, Constantinos S. Kyriakis

**Affiliations:** ^1^Department of Pathobiology, College of Veterinary Medicine, Auburn University, Auburn, AL, United States; ^2^Veterinary Faculty, University of Thessaly, Karditsa, Greece; ^3^Center for Vaccines and Immunology, University of Georgia, Athens, GA, United States

**Keywords:** animal health, infectious diseases, zoonotic diseases, next generation vaccines, vaccine technologies

Conventional vaccine technologies, such as inactivated and attenuated live vaccines, have saved millions of lives during the past century, yet the unmitigated spread of SARS-CoV-2 revealed a blind spot in our capacity to respond to emerging infectious diseases (1). Suboptimal performance issues, high costs and limitations in regards to scale-up production, illustrate the major caveats of traditional vaccine approaches for a time-sensitive response to emergent pathogens with pandemic potential (2, 3). Despite significant advances in vaccine research and development, human vaccinology was arguably kept in a relative stalemate in the pre-pandemic era with limited innovative vaccine approaches receiving licensure (4). On the other hand, veterinary science is a fertile ground for the development and commercialization of novel vaccine technologies. Direct evaluation of vaccine efficacy in target species capitalizes on the growing interest in livestock and companion animal health driving the progress and innovation of veterinary vaccines (3, 5).

The objective of this Research Topic was to bring attention on the state-of-the-art research conducted in veterinary vaccinology and highlight innovative vaccine technologies that are being explored and exploited for the improvement of animal health.

Aida et al. gave a comprehensive overview of the current advances in the field of veterinary vaccinology and reviewed commercially available novel vaccine technologies utilized in animal health, including recombinant protein/subunit vaccines, DNA constructs, viral vector technologies, and DIVA vaccines. This study reported that 52% of licensed novel vaccines in animal health were viral vector technologies, while subunit-recombinant protein vaccines were the second most available platform with 27%. Additionally, the vast majority of innovative veterinary vaccines are commercialized in food animals, with swine constituting approximately one third of the overall licensed novel vaccines.

Inactivated and attenuated live vaccines which represent first-generation vaccine technologies, are often reported to be less effective in inducing sufficient protection against a plethora of pathogens, including the porcine epidemic diarrhea virus (PEDV), a swine enteric coronavirus. Singh et al. developed a novel PEDV vaccine that utilized elements of both inactivated and attenuated live vaccines for the generation of an immunogenic construct that shows diminished, yet not abolished virus replication, which is a requirement for the elicitation of mucosal immunity and protection from PEDV.

A different coronavirus that causes significant economic losses to the poultry industry is the Infectious Bronchitis virus (IBV). While vaccines are the most effective countermeasure for disease prevention, limitations in vaccine effectiveness against heterologous IBV strains, pose a great threat in disease control. Improvement on current serological assays such as ELISA is pivotal for the development of more potent IBV vaccines. In this regard, Yin et al. developed and validated an ELISA that utilized a peptide comprised by a conserved, across variant IBV strains, epitope of the S2 subunit of the spike protein. The high sensitivity and specificity of this novel diagnostic assay facilitates early identification of anti-IBV antibodies from day 7 post-immunization and detection of antibodies against multiple IBV genotypes, and could prove to be a valuable tool for the generation of effective IBV vaccines.

Second-generation vaccine technologies, constituted by subunit and recombinant protein vaccines evoke antigen-specific immune targeting and represent an attractive alternative to the safety and production cost concerns of first-generation vaccines (6). Kalaiyarasu et al. recently optimized a recombinant M2-HA2 fusion protein, comprised by conserved regions of the corresponding M2 and Hemagglutinin (HA) proteins, across highly-pathogenic avian influenza virus (HP-AIV) strains, as a broadly protective HP-AIV vaccine strategy. However, a relative disadvantage of second-generation vaccine platforms, is that they often require the presence of adjuvants (7). Thus, an important aspect for the generation of effective vaccines is the enhancement of their immunogenicity by including a potent adjuvant. Lee et al. evaluated a novel adjuvant that was able to protect vaccinated mice from lethal AIV challenge and elicit comparable, to the commercial adjuvant, humoral responses against AIV and Newcastle disease virus in vaccinated chickens.

In addition to poultry, swine are a natural host of influenza A virus (IAV), which causes substantial economic impact to the pork industry. A review by Gracia et al. addressed the implications of utilizing commercial inactivated vaccines for disease control as a result of the complex epidemiology of IAV worldwide. Additionally, the authors provide an overview of the innovative IAV vaccine approaches currently explored in swine. First-generation IAV vaccines predominately target anti-HA specific epitopes and show limited efficacy against heterologous strains. Neuraminidase (NA), is an attractive IAV immunogen due to the limitations of HA-targeting constructs. Anti-NA antibodies inhibit the enzymatic activity of NA thus rendering Neuraminidase Inhibition (NI) assays as the golden standard for assessing NA-targeting humoral responses (8, 9). Skarlupka and Ross reported that raw sera may have non-specific NA inhibitory activities. Innate NA inhibitory properties can skew the NI assay results, if sera is not properly treated, which could prove detrimental especially when assessing novel NA-based vaccine technologies.

Similar to viruses, intracellular bacteria (ICB) require the induction of both humoral and cell mediated responses for effective clearance, and oftentimes ICB infections are characterized by the evasion of the former (10). On this account, Kim et al. measured the differential antibody responses induced by different antigenic forms of Salmonella Gallinarum (SG) in vaccinated chickens, with the intention of detecting antigenic epitopes that could be utilized in highly immunogenic SG vaccines. Another ICB infection that poses a significant zoonotic threat is Brucellosis. Huy et al. developed and evaluated the efficacy of a novel vaccine consisting of four recombinant Brucella abortus proteins. This combined subunit vaccine (CSV) enhanced the expression of innate bactericidal factors and conferred comparable protection in mice against disease and bacterial replication, to the commercial vaccine, by inducing a robust Th1 phenotype immune response.

In summary, this Research Topic highlighted some of the latest developments and innovations in the dynamic field of veterinary vaccinology research. It is essential to keep exploring and investigating novel vaccine approaches if we aim to effectively control infectious diseases in public and animal health.

## Author Contributions

All authors listed have made a substantial, direct, and intellectual contribution to the work and approved it for publication.

## Conflict of Interest

The authors declare that the research was conducted in the absence of any commercial or financial relationships that could be construed as a potential conflict of interest.

## Publisher's Note

All claims expressed in this article are solely those of the authors and do not necessarily represent those of their affiliated organizations, or those of the publisher, the editors and the reviewers. Any product that may be evaluated in this article, or claim that may be made by its manufacturer, is not guaranteed or endorsed by the publisher.
